# Study on the Function of Conveying, Kneading Block and Reversing Elements on the Mixing Efficiency and Dispersion Effect inside the Barrel of an Extruder with Numerical Simulation

**DOI:** 10.3390/foods12183503

**Published:** 2023-09-20

**Authors:** Min Wu, Dongyu Sun, Tong Zhang, Chengyi Zhou, Bowen Zhang

**Affiliations:** College of Engineering, China Agricultural University, No. 17 Qinghua East Road, Haidian District, P.O. Box 50, Beijing 100083, China

**Keywords:** extruder screws, numerical simulation, mixing degree, shearing rate, particle tracking

## Abstract

In order to better understand the extrusion process mechanism of plant protein inside a barrel, the parameter changes and flow characteristics of fluids under conveying, kneading block and reversing elements were investigated with numerical simulation. The results showed that the shear rate increased obviously with the increase in pitch; the shear rate value of the reversing element was larger, while that of the kneading block was the opposite. The screw combinations of conveying, kneading blocks and reversing elements all have a certain degree of mixing effect on the particles, and the reduction in pitch can effectively increase the mixing effect of the particles. The conveying element can provide a relatively constant acceleration for the particles, due to the pumping capability and pressure buildup as the pitch increases. The kneading block and the reversing element can increase the leakage flow between the discs and backflow, resulting in an extension of the residence time distribution that facilitates fluid interaction in the barrel and improves the dispersion of the particles. The restraint by the reversing element on the particles is obviously weaker than that of the kneading block and shows a higher particle mixing degree. Overall, the influence of different elements on the flow condition, mixing degree and residence time is significantly different, which improves the process controllability and provides references for potential applications to meet multiple demands.

## 1. Introduction

With the increasing popularity of plant-based foods on the market, coupled with the demand for sustainable green development, the application of extruders has been widely expanded due to the fact that more functions can be achieved, including conveying, crushing, mixing, melting, shearing, chemical reactions, drying and liquid–solid separation [1]. The procedure of extrusion can be generalized into two stages, namely mixing and cooking, and the physical and chemical properties of the food ingredient change, such as the destruction of starch particles, crystal melting, protein unfolding, cross-linking and re-aggregation, and dietary fiber de-polymerization, depending on the mechanical stress and heating effect generated by the space between the screw and the barrel. Moreover, with the different mixture of moisture added, proteins expand along the molecular chain and present a certain viscosity, and can be divided into low and high moisture extrusion, respectively [2]. The sudden release of pressure and the burst of hot water at the exit of the die promote the formation of a loose and porous structure in the extruded products. At present, it has been widely used in grain puffing, sustainable meat production and other fields [3,4].

Most of the current research focuses on the regulation of the extruded product by varying the extrusion operating parameters, such as the extrusion temperature, the screw speed, the moisture content in the barrel and so on [5,6,7,8]. To obtain a better extrusion effect, some studies have also further improved the quality characteristics of the extrudates by adding other components of raw materials. Immonen, M et al., investigated the de-starched oat and pea protein blend under high moisture extrusion [9]. The component and concentration of the protein isolate, and different starch types, can affect the texture properties of the extrudate [10,11]. However, they ignored the fact that the mixing efficiency can also directly affect the final product’s properties, and the changes in the parameters of the extrusion operation can also affect the flow of the fluid inside the barrel [12]. This is due to the fact that the arrangement and combination of different screw elements inside the barrel will directly affect the shearing and mixing effects [11]. In addition to determining the form of the fluid channel, the geometry of the screw element also impacts the direction and strength of the pressure flow at a specific flow rate and speed. High mixing capacity, good pressure accumulation, better flexibility and a large application range of the extrusion process can be obtained by the free combination and splicing of various types of screw elements [13,14].

Transportation of the fluid inside the extruder, mixing of the components and the residence time of the melt inside the extruder can be influenced by the combination of the screw elements (conveying, engaging and reversing elements) [15]. Changes in the pitch, stagger angle, length and location will affect the screw configuration and the entirety of the function of the screw, which further changes the quality characteristics of the extrudate [16]. Improper modifications can also have adverse results, such as browning of the product caused by a prolonged residence time, which intensifies the Maillard reaction during extrusion [17]. Hertel et al., also found that extending the mixing time or higher rotation speeds can significantly reduce the fine particle fraction [18]. Therefore, proper adjustment of the screw elements can effectively solve the problems of poor formability and a different universality in the extrusion process. As the main component of the screw, the conveying element promotes material flow along the screw, and the generation of axial propulsion pressure. The conveying capacity of this element increases with the increase in pitch, so the conveying element is normally placed in the feeding area and the melting area of the extruder to ensure the flow and compression of the bulk material [19]. Compared to the conveying element, the misalignment angle between the discs in the kneading block greatly improves the mixing shear degree of the fluid, but also weakens the conveying capacity [20,21]. Zhang et al., found that local flow variations caused by the kneading block increased the mean residence time and mixing capacity, and the mixing degree increased with the increasing misalignment angle [12]. In addition, the high pressure area generated by the kneading block forces the fluid backflow, and its degree increases with the thickening of the kneading block [22]. The reversing element prevents the original flow tendency by opposing screw flight, which also promotes the mixing of the fluids and energy input. Studies have shown that the use of a reversing screw element can increase the degree of starch degradation and the porosity of the extrudate compared to a kneading block [16,23]. Zheng, J also found conveying, kneading block and reversing elements have a great effect on the enzymatic digestibility of corncobs [24].

Water as a plasticizer in the extrusion process greatly affects the flow behavior of the fluid; the flow situation inside the extruder also depends on the highly coupled operating conditions, poly-condensation dynamics and rheology dynamics, due to changes in the interaction between the pressure flow and drag flow generated during the rotation of the screws [25]. Given that raw food materials have deformable, hygroscopic and amorphous properties in a multi-phase mixing and dispersion system, it is necessary to control the extrusion process through theoretical analysis in order to improve the adaptability of different process parameters and meet various needs in different application scenarios [26,27]. Under the limitation of the closed chamber in the extruder, numerical simulation can effectively reveal the shear mixing effect of the fluid, accurately describe the local flow characteristics inside the barrel and provide more detailed information about the “black box” than the experimental study.

Due to a lack of research on the structural and functional roles of the screw element, it is normal to find that the conveying element dominates most of the discussion on the screw inside the extruder, which reduces the universal application of various materials and processes in regard to the extrusion process and leads to less uniformity of the extrudate quality. The influences of the component types on the shear rate, particle acceleration effect, residence time and mixing action of the flow field are explored through the modeling and simulation of different screw components, and the functional characteristics of each screw component are further defined. The shearing and mixing effects of the screw configuration inside the barrel can be dynamically revealed by methods for image and particle tracking, which improves the process controllability of the extrusion, and provides theoretical support for the application of screw functional components in different fields.

## 2. Materials and Methods

### 2.1. The Establishment of the Screw Model

**Figure 1:**
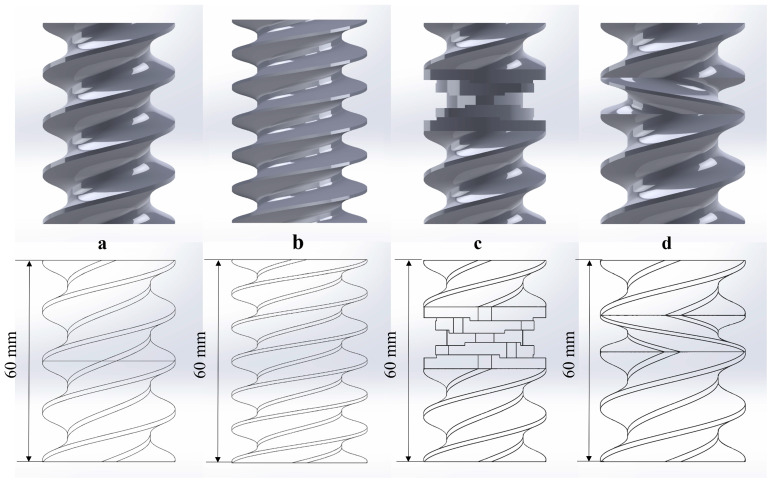
The screw model of different screw combinations: conveying (**a**,**b**), kneading block (**c**), and reversing elements (**d**).

A screw combination, 60 mm in length, made up of different configurations of conveying (C-A and C-B), kneading block (C-C) and reversing elements (C-D) was built using SolidWorks 2018 software (Wyman Street, Waltham, MA, USA), as shown in Figure 1.

C-A consists of two SE 30/30/R pieces; C-B consists of three SE 20/20/R pieces; C-C consists of two SE 20/20/R pieces and a kneading block of KP 45/5/20/R; and C-D consists of an SE 30/30/R, an SE 20/20/R, and a reversing element of SE 20/10/L.

### 2.2. Material Selection and Screw Model Setup

Water and non-Newtonian fluids were used as the media of the flow field to test the shear capacity of different screw configurations. For the non-Newtonian fluids, the consistency index (m) was set to 565 Pa∙s and the flow characteristic index (n) was set to 0.26, which were obtained through the fitting curve of the soybean protein viscosity–shear rate according to the power law model, with an R^2^ of 0.997. The base point in the rotation axis was set at the center bottom of the screw, and the dynamic grid of the rotation domain was established with the *Y*-axis for the rotation axis. The rotation speed of the screw was set at 100 rpm. Gravity was applied to the screw in the Z direction, and the effect of the polymer flow mass was also considered.

The physical model of the flow was set as incompressible flow, which ignored the inertial term, and the fluid flow equation is shown below:

(1) $$\rho \frac{\partial u}{\partial t}=\nabla \cdot (-pI+K)+F$$

(2) ρ∇⋅u=0

where *ρ* denotes the fluid density, *u* denotes the velocity component and *F* denotes the viscous force and external force,

For the Newtonian fluid:

(3) $$K=\mu (\nabla u+{\left(\nabla u\right)}^{T})$$

where *K* denotes the viscous stress tensor of the fluid.

For the non-Newtonian fluids, according to the power law model [28]:

(4) $$\mu =m{\left(\frac{\dot{\gamma }}{{\dot{\gamma }}_{ref}}\right)}^{n-1}$$

(5) $$\dot{\gamma }=max(\sqrt{2S:S},{\dot{\gamma }}_{\min})$$

(6) $$S=\frac{\left[\nabla u+{(\nabla u)}^{T}\right]}{2}$$

where *μ* denotes the dynamic viscosity, *γ* denotes the shear rate, *m* denotes the fluid consistency coefficient and *n* denotes the flow behavior index.

The boundary wall condition was set as no slip, that is, the fluid velocity at the wall is consistent with the fluid velocity:

(7) $$u=u_{tr}$$

The inlet of the inflow velocity was set at 5 mm/s, and the outlet of the static pressure was 0 pa. Finally, 2,313,505 mesh cells were formed with an average mesh mass of 0.68. The positive direction of the *Y*-axis is regarded as the extrusion direction. Two points (0, 0, 20) and (0, 60, 20) are used as a three-dimensional spline to obtain the shear velocity of the fluid.

### 2.3. Fluid Flow Particle Tracking the Physical Field

The mixing efficiency of the different screw elements were tested, and the fluid flow particle tracking the physical field was added to study the particle motion trajectory, particle velocity, residence time distribution and other indicators. The corresponding physical quantity equation for fluid particle tracking is shown as Equation (8):

(8) $$\frac{dq}{dt}=u+\frac{\tau_{p}}{m_{p}}F_{ext}$$

where **q** denotes the particle position vector, *m_p_* denotes the individual particle mass, F*_ext_* denotes the external force, and *τ_p_* denotes the constant time and can be calculated according to Equation (9):

(9) $$\tau_{p}=\frac{\rho_{p}d_{p}^{2}}{18\mu }$$

Since the particle velocity response time *τ_p_* is much smaller than the simulation solution time range, and the correlation role of inertia in particle motion should be ignored, the fluid type is selected as Newtonian, and the fluid inertia term is ignored.

The initial velocity of the particle was set as 0 to make sure the particle’s velocity changed with the velocity of the fluid. The number of released particles was 3000, the particle density was 2200 kg/m^3^, and the particle size was 5 × 10^−7^ m. The particle release location was at the entrance, and the velocity of the particles did not change after they impacted the wall.

(10) $$V=V_{c}$$

The physical field of the drag force (**F***_D_*) is set in the whole fluid domain and can be determined by the properties of the particle velocity response time, mass, velocity and fluid viscosity. The relevant physical field is shown in Equation (11).

(11) $$F_{D}=\frac{1}{\tau_{p}}m_{p}(u-v)$$

Moreover, the particles are also subject to mass forces (**F***_vm_*) and pressure gradient forces (**F***_p_*), which can be calculated according to the following equations:

(12) $$F_{vm}=\frac{1}{2}m_{f}\frac{d(u-v)}{dt}$$

(13) $$m_{f}=\frac{1}{6}\pi d_{p}^{3}\rho$$

(14) $$\frac{d(u-v)}{dt}=\frac{\partial (u-v)}{\partial t}+v\cdot \nabla (u-v)$$

(15) $$F_{p}=m_{f}\frac{Du}{Dt}=m_{f}(\frac{\partial u}{\partial t}+u\cdot \nabla u)$$

where *m_f_* denotes the fluid mass replaced by the particle volume and *D*/*Dt* denotes the material derivative in the direction of the fluid velocity.

Since the density of the particle phase is close to that of the fluid phase, the virtual mass force and pressure gradient force may be close to the same order of magnitude as the drag force and cannot be ignored in the calculation.

Physical quantities, such as the residence time distribution function, cumulative distribution function, average residence time and variance, are obtained through simulation calculations. The residence time of the particles can characterize the time that different parts of the mixture spend in the container [29], and the function *E*(*t*) can be expressed as Equation (16):

(16) $$E(t)=\frac{C(t)}{\int_{0}^{\infty }C(t)dt}$$

The number of fractions spent by tracer species leaving the container, between *t*_1_ and t_2_ inside the container, can be defined as:

(17) $$\delta_{t_{1}-t_{2}}=\int_{t_{1}}^{t_{2}}E(t)dt$$

The residence time step ΔT was set as 0.2 s, and the ending time was set as 20 s to calculate the residence time distribution of the particles in the barrel.

The data on the position of the output particle in the three-dimensional coordinate system, namely the particle speed, pressure and other parameters were collected at y = 0, y = 20 mm, y = 40 mm, y = 60 mm and y = 80 mm to observe the trajectory of the particle changes. A transient solver was used to observe the movement of the particles in the barrel at different time steps, and the relative tolerance was controlled at 0.001.

### 2.4. Thin Matter Transfer in a Physical Field Setting

The fluid deformation under the action of the screw inside the barrel can be regarded as a dilute material transfer process. When considering the mass transfer of a component in a solute or gas mixture, it is necessary to note that the concentration gradient causes diffusion. During the overall fluid motion, convection makes a flux contribution to the chemicals. Therefore, the combined effects of convection and diffusion need to be analyzed.

For dilute matter:

(18) $$N_{i}=-D_{i}\nabla c_{i}+c_{i}u$$

(19) $$\frac{\partial c_{i}}{\partial t}+\nabla \cdot N_{i}=R_{i}$$

where *N_i_* denotes the molar flux, *D_i_* is 10^−9^ m^2^/s which denotes the diffusion coefficient and *c_i_* denotes the concentration.

Changes in the screw geometry can increase the surface contact area between different fluid layers and reduce the separation length between these layers. Although convection can significantly shorten the time scale of diffusion, it is still diffusion that produces mixing. The particle inlet was considered to have a fully developed laminar flow. The dimensionless concentration ranged from 0 to 1, with 0.5 representing a completely mixed solution, and 0 and 1 representing a mixture that does not mix at all after screw action.

**Figure 2:**
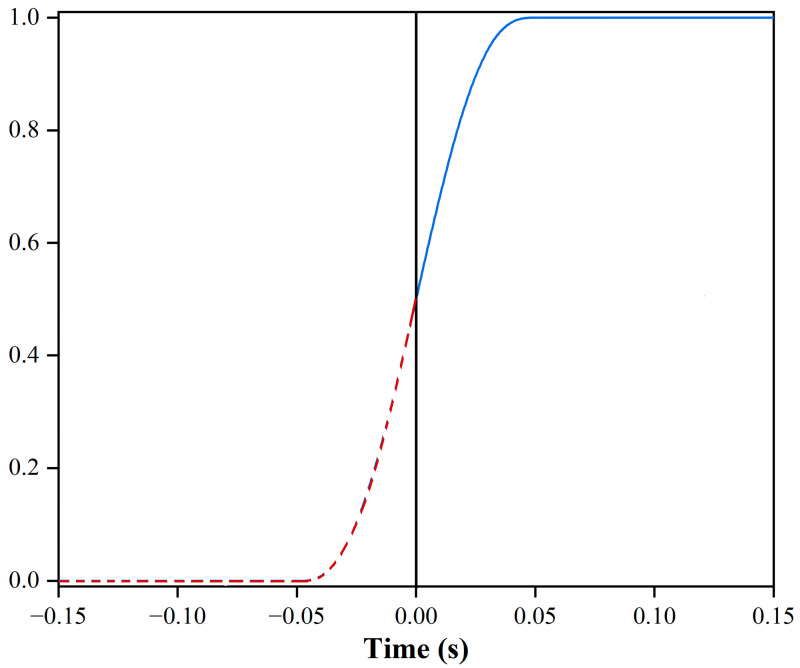
The step function of different screw combinations at the inlet (The dash line is the function which did not participate in the simulation phase, and solid line is the function that participate in the simulation phase).

The inflow concentration is set as the product of the inlet concentration and the step function, which can facilitate the convergence of the calculation results. The inlet concentration is set at 1 mol/m^3^, and the step function is consistent with the initial conditions for the velocity in the model, starting from zero, as shown in Figure 2.

The concentration changes at y = 0, y = 20 mm, y = 40 mm, y = 60 mm and y = 80 mm were collected in the XZ cross-section to calculate the concentration change curves at different locations.

## 3. Results

### 3.1. Analysis of the Flow Field Shear Rate of the Screw with Different Configurations

**Figure 3:**
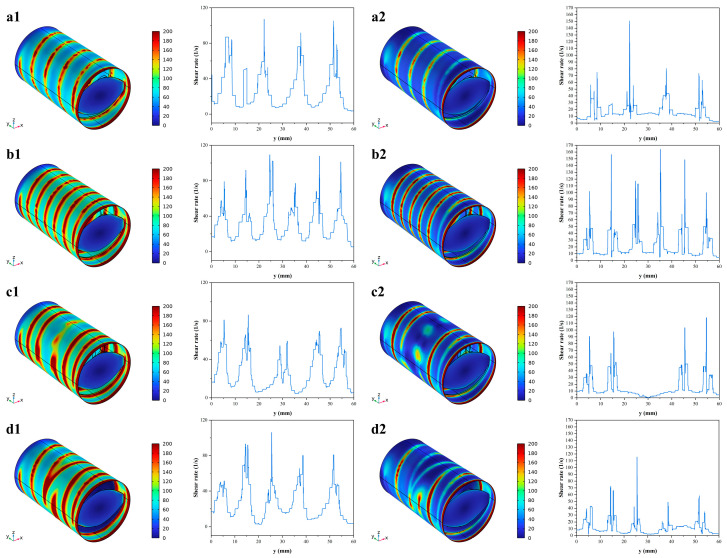
Shear rate contour map of fluid in the barrel under different screw elements ((**a1**,**a2**) C-A, (**b1**,**b2**) C-B, (**c1**,**c2**) C-C and (**d1**,**d2**) C-D).

The shear rate distribution of four screw element configurations for Newtonian fluids (1) and non-Newtonian fluids (2) is shown in Figure 3, and the contour maps for them appear red at the screw flight and blue at the screw channel. This was due to the fact that the larger rotation radius of the screw increased the linear velocity, which resulted in a higher shear rate; the sharply reduced distance between the screw flight and the inner barrel also provides potential basic conditions for fully mixing the materials and changes in the protein structure. This is consistent with the results from Sun et al. [30]. It can be seen that the shear rate distribution of screw elements with different pitches was obviously different, and a pronounced periodicity fluctuation in the shear rate was shown with the decreasing pitch, which effectively improved the stability of the shear rate, and had little effect on the shear rate value (Figure 3(a1,a2,b1,b2)). While the shear rate of protein increased obviously with the increase in the pitch: the majority of the shear rate peaks for the 30 mm pitch in C-A were mainly distributed in 70~80 s^−1^ (Figure 3(a2)), and the shear rate mainly fluctuated between 100 and 160 s^−1^ for the 20 mm pitch (Figure 3(b2)). Moreover, the shear rate distribution range of the non-Newtonian fluids increased significantly, which indicated that the viscosity change in the fluid also has a great influence on the shear rate. The shear rate of the non-Newtonian fluids at the kneading block kept within a low shear rate range (1~10 s^−1^), and its value at the front and rear elements also reduced from 70 s^−1^ to 25~30 s^−1^. This may be due to the coupling effect of drag flow, disc cross flow and pressure flow that reduced the fluid velocity, increased the space between the screw and the barrel, and extended the fluid residence time [31]. The reversing element showed a larger shear rate value, which can be ascribed to the backflow from the reversing element leading to a surge in the shear rate of the fluid. On the other hand, it is also related to the sharp angle of the screw connection between the forward and reversing elements. The encounter between the reflux and the front-end fluid greatly reduced the flow rate and shear rate of the latter, while the fluid at the back end lacked effective propulsion and significantly reduced the shear rate. Therefore, the reversing element has a great effect on reducing the shear rate before and after the element.

### 3.2. Analysis of Particle Dispersion and Panglais Cross-Section of the Screw with Different Configurations

**Figure 4:**
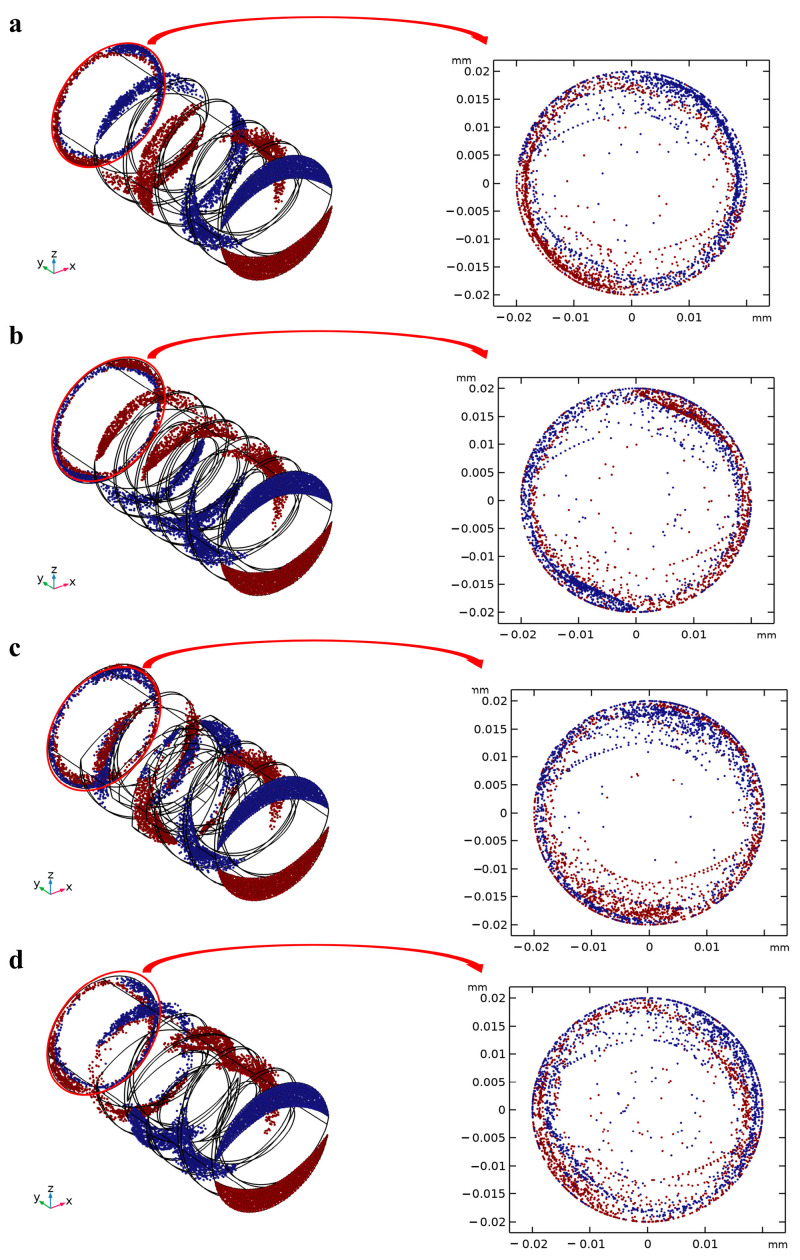
Panglais cross-section and phase diagram of the screw with different configurations ((**a**) C-A, (**b**) C-B, (**c**) C-C and (**d**) C-D).

The Panglais diagram can transform the continuous particle motion process into a discrete cross-section by reducing the dimension of the movement process and preserving the topology of the particle motion at the same time. The diagram of particle motion at the ZX cross-section under different screw configurations is shown in Figure 4.

**Figure 5:**
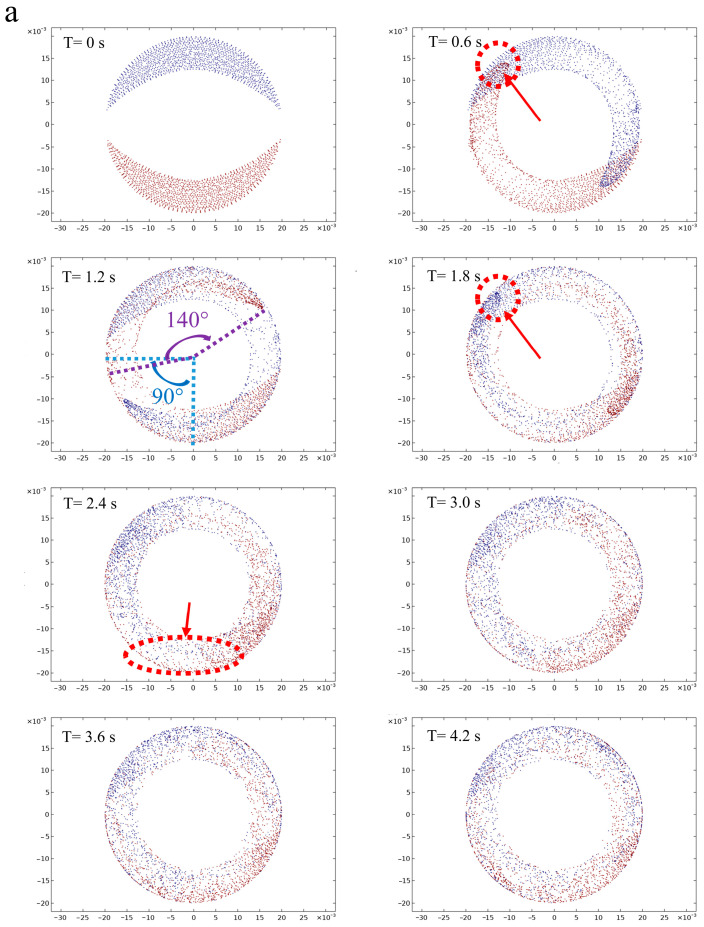

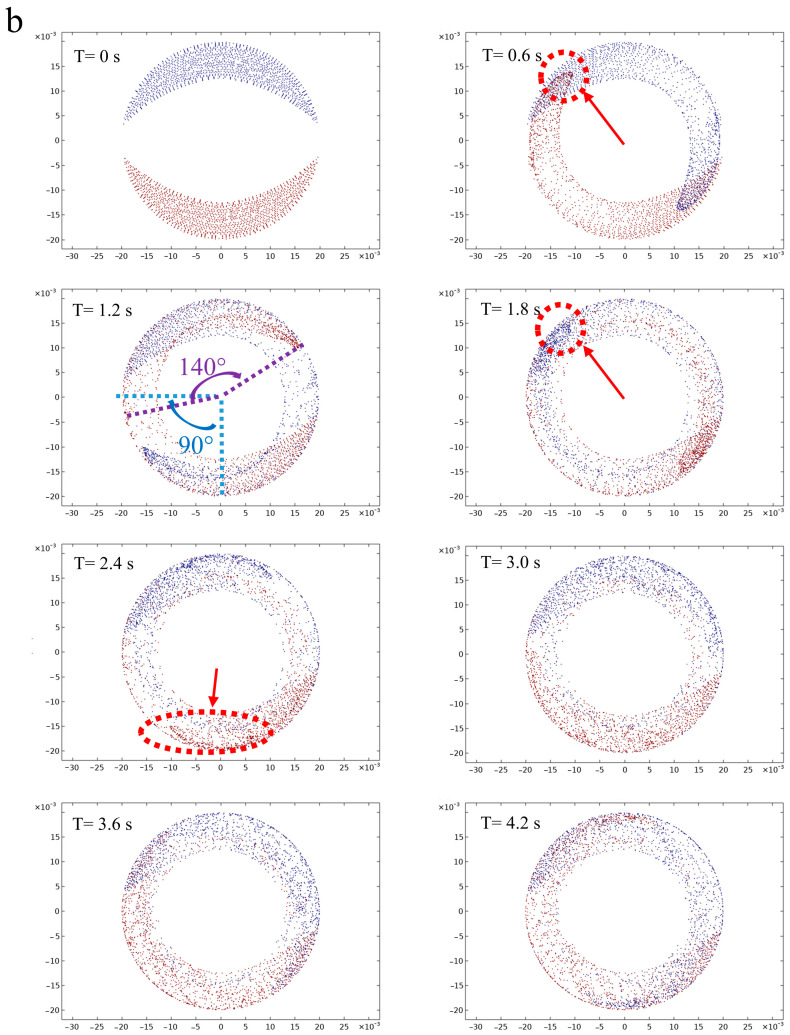
Phase diagram of the screw with the kneading block ((**a**) C-C) and reversing elements ((**b**) C-D) with different times.

In the initial state, the blue particles were distributed above the screw and the red particles below the screw, which were gradually mixed after shearing for all four screw configurations, meaning that the screws can promote the mixing degree of the particles. Driven by the conveying element, most of the particles still flowed along the rotation of the screw, which weakened the dispersion effect of the particles, leading to a relatively clear and center, symmetrical distribution of the red and blue areas on the end section of the screw. This illustrated that the particles were trapped in the barrel under different particle velocities, due to the mixing of unidirectional conveying elements, which mainly relied on drag force and pressure gradient force. Compared with C-A, C-B reduced the pitch and increased the number of screw channels in the second stage, which was conducive to the generation of velocity differences for more particles and strengthened the mixing effect to a certain extent. Moreover, the movement trajectory of most particles under the conveying element is fitted with the inner surface of the barrel, and the introduction of the kneading block and the reversing element effectively improved the dispersion of the particles. Therefore, the distribution of particle motion states changing with varying amounts of time under the kneading block (C-C) and reversing elements (C-D) was further explored and is shown in Figure 5. It can be seen that the fluid particles were subjected to different degrees of shear and stress with the passage of time.

In the initial stage, part of the particles moved along the screw channel and formed a unidirectional circumferential shear flow under the drive of the conveying element, which gradually began to disperse and presented a “crescent moon” shape. This was mainly due to the fact that some of the particles near the screw flight obtained a larger velocity than the particles at the screw channel, while the particles at the tail showed a stalling phenomenon. This scenario was intensified at 1.2 s, in which the head particles had turned by about 140 degrees, while the middle particles had only turned by about 45 degrees. When the time reached 1.8 s, the originally sharp head particle swarm gradually presented as rounded corners, which is due to the particles entering the kneading block area. Part of the head particles were allowed to pass through the misalignment angle between two sequential discs, and the rest of them were accumulated between the two layers of kneading blocks, eventually slowing down some of the head particles (Figure 5a) [32]. A bifurcation flow was formed after the shear mixing of the kneading block, due to the fact that part of the fluid was pushed out to the adjacent disk area, which reduced the trend in the head particle movement and significantly increased the distribution degree of the red and blue particles. The mixing degree of the blue and red particles showed a uniform distribution at 4.2 s compared to the initial state, indicating that the particle mixing degree can be improved under the kneading block [20]. The reversing element can force the particles near the screw channel to flow back and collide with the particles at the front, effectively inhibiting the flow of particles, but having little impact on the velocity of the particles close to the barrel. This can also be demonstrated by the fact that when the particles pass through the reverse cell for 1.8 s, a portion of the particle located inside was obstructed, and the mixing degree of red and blue particles was further increased by the action of the reversing elements, which can be observed in 4.2 s. Jun Zheng et al., also found that the reversing element can promote the redistribution of the cellulose fibers [24]. It is worth noting that the inhibition effect of the particle reversing element was obviously weaker than that of the kneading block. From the phase diagram for 2.4 s, it can be seen that the progress of a large number of particles under the reversing element was significantly faster than that of the kneading block, indicating that the kneading block can effectively extend the residence time of the particles inside the barrel and eventually obtain a better mixing and dispersion effect.

### 3.3. Analysis of the Particle Velocity Variation of the Screw with Different Configurations

**Figure 6:**
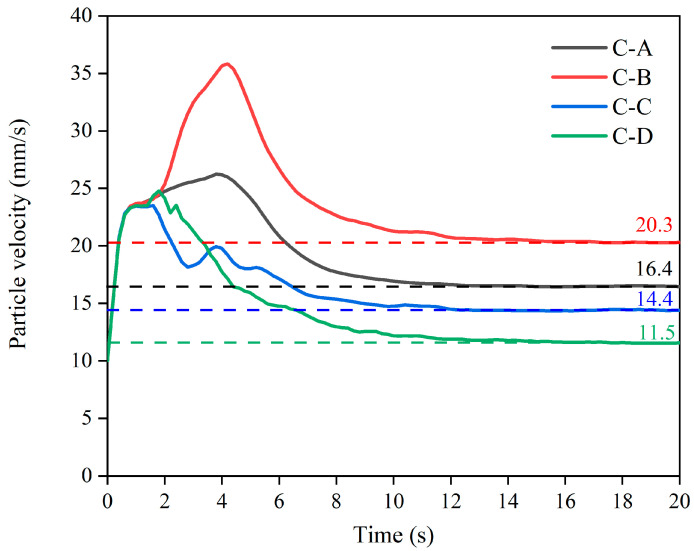
The particle velocity of the screw with different configurations for different times.

The particle velocity changes inside the barrel under four screw component configurations are shown in Figure 6. The velocity of the particles first increased and then gradually decreased under the extrusion process, finally exiting the barrel with a relatively constant velocity with a velocity order of C-B > C-A > C-C > C-D. The velocity curves of all the particles under the different screw types overlapped in the initial stage (within 0–0.8 s) until the velocity reached 23 mm/s, which was due to the similar velocity values of the particles before the screw effect. The curve of C-A was composed of two identical conveying elements, and the particle climbed to about 26 mm/s at a relatively stable rate after the speed reached 10 mm/s, indicating that the conveying element provided a relatively constant acceleration for the particle. The velocity value of C-B showed a similar upward trend to that of C-A, and then rose to 36 mm/s, indicating that a decreasing pitch is helpful to improve particle acceleration. Moreover, the velocity of both of them peaked at about 4 s, indicating that the residence time of large particles inside the barrel is similar under the conveying elements in the two configurations. The particle velocity of C-C reduced after reaching a peak, accompanied by four peaks that gradually declined. This can be attributed to the increased interleaving angle of the kneading block, which reduced the positive pressure gradient. The shearing effect of five meshing pieces gradually weakened the mixing and flow of the particles, which fluctuated the particle velocity and gradually decreased the variation amplitude, finally stabilizing at 14.4 mm/s. The particle velocity of the C-D curve showed an obvious “spike” under the action of the reversing element, which was related to the surge in fluid velocity caused by the extremely small area at the intersection of the forward and reversing elements. The outflow velocity of C-C and C-D leveled off after 14 s and stabilized at 14.4 mm/s and 11.5 mm/s, respectively, indicating that the kneading block and the reversing element can obstruct the movement of the particles inside the barrel to some extent, providing a certain basis for a better mixing effect [26].

### 3.4. Analysis of the Particle Residence Time of the Screw with Different Configurations

**Figure 7:**
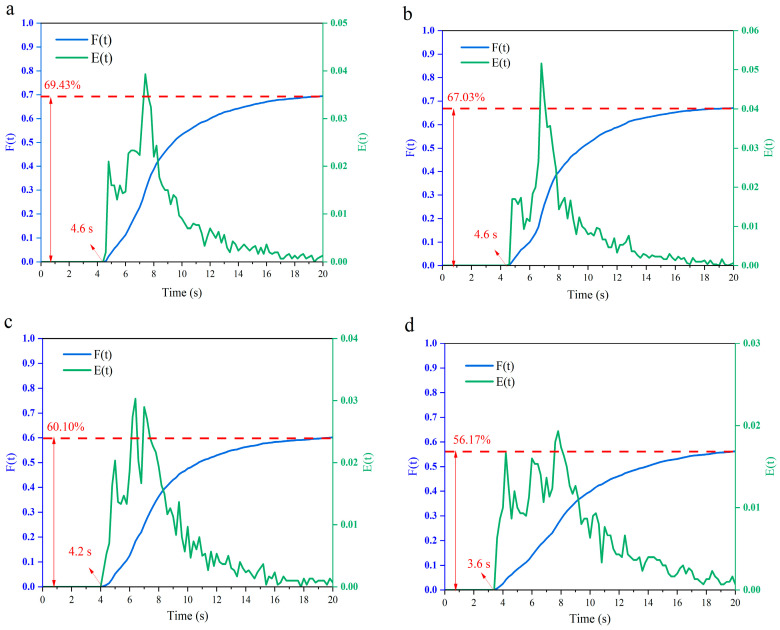
Particle residence time distribution inside the barrel for different screw configurations ((**a**) C-A, (**b**) C-B, (**c**) C-C and (**d**) C-D).

The particle residence time distribution in the flow field under different screw configurations is shown in Figure 7. It can be seen that the width of the distribution becomes narrower over time [33]. The particles began to flow out of the barrel at 4.6 s for screws C-A and C-B, and the peak of the *E(t)* curve of C-B was 5% higher than that of C-A, indicating a higher instantaneous outflow of particles for small pitches. This is due to the fact that the smaller pitch provided a larger flow speed for the particles, which significantly reduced the residence time of the fluid in the barrel, which is consistent with the conclusion that C-B has a higher particle velocity in Section 3.3. However, the cumulative outflow rate of C-B was only 67.03%, which was significantly lower than that of C-A (69.43%), due to the stronger pumping capability and pressure built up with a large pitch [34]. This is in accordance with the research results by Pan et al. [35]. Compared with the conveying element, the *E(t)* curve of the kneading block appeared at 4.2 s, which can be ascribed to the leakage of flow inside the barrel. The exhibition of two similar “peaks” indicated that the particle dispersion inside the barrel increased significantly, and was no longer concentrated in a certain time step, and each particle fraction (2.8~3%) was lower than that of the delivery element (4~5%). This can be attributed to the staggering of the kneading block, which increased the leakage flow between the discs, which increased the backflow of fluid, resulting in a large residence time distribution that facilitated fluid interaction in the barrel. This is consistent with the work of Sarhangi Fard et al. [32], who mentioned that the interleaving of two sequential discs promoted the exchange of fluid between adjacent screw channels, compared to the conveying element. Therefore, the positive axial transport volume was reduced due to the generation of backflow when interleaving was increased. Although the first particle had the shortest time to flow out of the barrel (3.6 s) for the reversing element, its instantaneous outflow molecular rate was the lowest (1.7%), indicating that the reversing element can promote most of the particles to flow inside the barrel without flowing out. The appearance of the reversing element also resulted in a pressure drop in the barrel, which prolonged the residence time of the particles and finally facilitated the reaction [36]. Moreover, the multiple peaks in the *E(t)* curve also indicated that the particles were thoroughly mixed inside the barrel under the reversing element, which is consistent with the previous findings (Section 3.2).

### 3.5. Analysis of the Particle Concentration Variation of the Screw with Different Configurations

**Figure 8:**
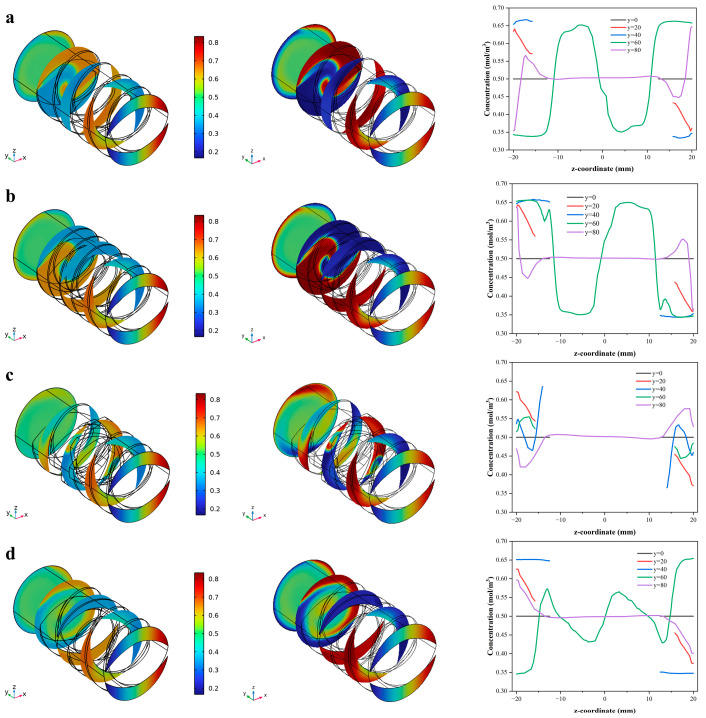
Particle residence time distribution in the barrel for different screw configurations ((**a**) C-A, (**b**) C-B, (**c**) C-C and (**d**) C-D).

The dimensionless concentration changes along the extrusion direction under different screw configurations are shown in Figure 8. The best value can be obtained at 0.5. 

The profile in all cross-sections showed that the mixing effect was weaker near the screw flight and better at the screw channel, and the mixing degree gradually increased with the extrusion process. The dimensionless concentration of C-A and C-B is similar, and the color distribution is basically the same. The concentration was around 0.6 and 0.4 at the y = 60 mm plane, indicating a relative lower mixing effect of the conveying element. Moreover, the concentration curve of the y = 80 mm plane indicated that the extreme mixing concentration for both C-A and C-B fluctuated around 0.66 mol/m^3^ and 0.34 mol/m^3^, respectively, indicating that the change in pitch does not significantly affect the concentration distribution, and that the appropriate extension of the screw length is conducive to improving the mixing effect. For kneading blocks and reversing elements, the concentration curve at the y = 80 mm plane is closer to 0.5 mol/m^3^ than that of the conveying element, which ranged from 0.44 to 0.56 mol/m^3^ and 0.42 to 0.58 mol/m^3^, respectively. This indicated that the particle mixing degree was more complete, and the backflow between the adjacent discs in the kneading block increased the cross-mixing particles in the cross-section, which eventually led to a more uniform mixing effect [34]. In addition, the mixing curve at the plane y = 20 mm was closer to 0.5 mol/m^3^ than that at the plane y = 40 mm in Figure 8d, indicating that a large number of particles can be mixed due to the formation of backflow with the reversing element.

## 4. Conclusions

The results showed that the shear rate at the edge of the conveying element was the largest, and a reduction in the pitch can reduce the shear rate and effectively accelerate the velocity of the particles with little effect on the fluid concentration. Kneading blocks can enhance the effective dispersion of particles inside the barrel, promote particle mixing, and improve stagnation and aggregation of most particles, while reversing elements can force the particles to flow back and collide with the front-end particles, effectively changing the flow state of the particles, and promoting the particles to flow inside the barrel without flowing out, eventually increasing the residence time of the particles. In this paper, the underlying motion patterns of the fluid inside the barrel under different screw elements was explored, which improved the process controllability of extrusion, and provided a theoretical basis for the potential application of screw functional elements in different food industries. However, numerical simulations still have limitations in terms of their representativeness of real-world conditions, hence, in the future, more experimental studies should focus on the quality attributes of extrusion products under different screw combinations, which can provide more information for the optimization of the extrusion process.

## Data Availability

Data is contained within the article.
